# Impact of Lower-Limb Asymmetries on Physical Performance Among Adolescent Female Tennis Players

**DOI:** 10.3390/life14121561

**Published:** 2024-11-28

**Authors:** Nagore Moreno-Apellaniz, Oscar Villanueva-Guerrero, Víctor Emilio Villavicencio-Álvarez, Santiago Calero-Morales, Elena Mainer-Pardos

**Affiliations:** 1 Health Sciences Faculty, Universidad San Jorge, 50830 Zaragoza, Spain; namoreno125@gmail.com (N.M.-A.); ovillanueva@usj.es (O.V.-G.); epardos@usj.es (E.M.-P.); 2 Department of Humans and Social Sciences, Universidad de las Fuerzas Armadas-ESPE, Quito 171103, Ecuador; victoremiliovillavicencio@gmail.com

**Keywords:** racket sport, asymmetries, jump, change of direction, speed, performance

## Abstract

This study aimed to determine the correlation between interlimb asymmetries and physical performance metrics in adolescent female tennis players. Methods: Twenty-five female tennis players (age: 13.29 ± 0.98 years; weight: 52.52 ± 7.31 kg; height: 162.91 ± 6.02 cm) consented to participate in this study. Their performance was evaluated using various variables, including bilateral and unilateral countermovement jump (CMJ), bilateral and unilateral horizontal jump (HJ), 180° change of direction (180COD) conducted with both the right and left legs, and a 20 m sprint. The systematic bias was evaluated by one-way repeated measure analysis of variance, Pearson’s correlation test for relationships between variables, and the kappa coefficient for consistency in the asymmetrical direction. Results: Significant relationships were found between CMJ asymmetry and the variables HJR (r = −0.47) and HJL (r = −0.44). In addition, significant relationships were found between HJ asymmetry and the variables CMJR (r = −0.60) and CMJL (r = −0.54), HJR (r = −0.64), HJL (r = −0.67), CMJ (r = −0.55), and HJ (r = −0.52). Conclusion: Jumping tests are important indicators of performance loss in female tennis players. In addition, no significant correlation was found between the 180COD and performance tests, suggesting that asymmetries in COD do not affect jump performance or speed. Based on these results, it is recommended to integrate individualized programs for each athlete to reduce asymmetries.

## 1. Introduction

Tennis is a sport characterized by repetitive unilateral movement patterns and high-intensity effort in which limb asymmetries play an important role [1,2,3]. A lack of balance between the limbs or muscle groups is called interlimb asymmetry [4]. Asymmetries can be caused by repetitive asymmetrical movements [5], injuries, or anatomical asymmetries [6]. During a tennis match, players typically run between 8 and 15 m and perform an average of 3–4 changes of direction (CODs) at each point, alongside other high-intensity actions, such as accelerations, decelerations, and jumps [1].

Recent studies have suggested that interlimb asymmetries may negatively affect the performance of high-intensity tasks, particularly those involving CODs [7,8]. In this regard, one study found that jumping asymmetries were associated with decrements in sprint and jump performance among elite youth athletes [9]. Furthermore, this study suggested that an asymmetry of 12.5% during a single-leg countermovement jump (SLCMJ) is related to reduced acceleration in young female soccer players [10]. Asymmetries between 10 and 15% can also increase the risk of injury [11]. The most common injuries for tennis players are the lower limbs (39–59%), followed by the upper limbs (20–40%) and trunk (11–30%) [12,13]. Therefore, it is important to control asymmetries in tennis players to mitigate injury risks.

On the other hand, a systematic review and meta-analysis showed that asymmetries appear to present a weak negative impact on COD and sprint performance, but not on vertical jumping [14]. Raya-Gonzalez et al. also concluded that there was not a significant relationship between interlimb asymmetries and physical performance measures, at least in elite youth soccer players [15]. Despite findings suggesting that asymmetries may have a minor negative impact on COD and sprint performance, it is necessary to consider the different contexts within various sports [16]. The absence of a significant correlation in some studies could imply that other factors, such as individual technique, muscle strength, and neuromuscular adaptation, may play a more critical role in performance than asymmetries themselves. Therefore, although some studies have indicated that asymmetries may not substantially affect all aspects of sport performance, it is essential to investigate this phenomenon across different sports, such as individual ones, to achieve a more comprehensive understanding.

Bailey et al. observed that females produced significantly greater interlimb asymmetries than males in the unilateral and bilateral jump variables [17]. In addition, the researchers concluded that weaker athletes displayed more asymmetry than stronger athletes. This suggests that addressing strength imbalances may help to reduce performance asymmetries in female athletes [2]. Neuromuscular training programs seem to be an optimal tool for reducing asymmetries, according to Madruga-Parera et al. [7].

In male tennis players, Villanueva-Guerrero et al. found significant negative relationships between CMJ and COD asymmetry with unilateral HJ variables (r = −0.30 to −0.53). In addition, CMJ asymmetry showed a significant relationship with CMJR (r = 0.49) and COD180R (r = 0.29), and COD asymmetry showed a significant relationship with COD180L (r = 0.40) [18]. Moreover, other recent research found significant negative relationships between CODS asymmetry and SLCMJ performance in both limbs (r = −0.50; r = −0.53) and CODS performance in both limbs (r = 0.50; r = 0.63) [7,9,11]. Therefore, interlimb asymmetries during CODS were associated with reduced performance in jumps and CODS tests, and CMJ and COD imbalances were associated with reduced performance in HJs.

Interlimb asymmetries have been shown to negatively affect performance in high-intensity tasks [16]. Jumping asymmetries have been associated with decrements in sprint and jump performance [9], as well as the risk of injury [19], particularly in sports requiring explosive power and agility. In the context of tennis, lower-limb asymmetries can influence performance by affecting the player’s ability to generate force, using explosive force generation tests such as countermovement jumps (CMJs), horizontal jumps (HJs) and the COD capacity that occurs in tennis as indicators [20,21]. Therefore, evaluating these asymmetries through standardized physical tests is critical to better understand their effects on the specific performance demands of tennis players. The present study used CMJ, HJ, and COD tests to assess these asymmetries, providing insight into how they relate to physical performance in tennis.

Some authors have analyzed the association between physical performance and asymmetries in male tennis players [7,9,11,18]. However, to the best of the authors’ knowledge, no such study has been conducted on female tennis players. Therefore, the aim of this study was to determine the correlation between interlimb asymmetries and physical performance variables in adolescent female tennis players.

## 2. Materials and Methods

### 2.1. Participants

Twenty-five adolescent female tennis players (age: 13.29 ± 0.98 years; height: 162.91 ± 6.02 cm; body mass: 52.52 ± 7.31 kg; body mass index: 19.75 ± 2.21 kg/m^2^) agreed to participate in this study. A preliminary power analysis was performed using G*Power software (version 3.1.9.3, from Düsseldorf, Germany) to determine the required number of participants. Given the study design, which examines variations within a single group, an effect size of 0.5, a significance level (alpha) of 0.05, and a desired power of 80%, we determined that 23 participants were needed. This study ultimately included 25 participants, yielding a statistical power of 84%.

Tennis players from two distinct academies participated in this study, all adhering to a structured training program that was consistent in both volume and methodology. This program comprised four 90 min sessions focused on technical and tactical skills, along with two sessions dedicated to physical training each week. During the data collection period, all players were injury-free. Written informed consent was obtained from the parents or legal guardians of all participants prior to participation. This study was approved by the University Ethics Committee (approval no. 46/2/22-23) and was conducted in accordance with the ethical principles of the Declaration of Helsinki (2013).

### 2.2. Procedures

The physical performance tests were carried out on a single day, at the usual time of training for the players (18:00), and in favorable weather conditions. Because these assessments are part of the Academy’s regular program, all players were already familiar with the procedures. Testing took place on a hard tennis surface with players wearing appropriate tennis shoes.

Before beginning the tests, players performed a warm-up protocol based on the Rise, Activate, Mobilize, and Potentiate (RAMP) system [22]. This was followed by three practice runs for each test to ensure test readiness. A 3 min rest period was provided between the final practice run and the start of the first test to optimize performance. The tests were performed in the following sequence: unilateral vertical jumps, bilateral vertical jumps, unilateral HJs, bilateral HJs, 180° change of direction, and a 20 m sprint. For unilateral tests, the right leg (dominant) was tested first, followed by the left leg (non-dominant). Between each race, a 3 min break was given to the players to allow them time to rest and hydrate.

#### 2.2.1. The Bilateral and Unilateral Countermovement Jump Tests

The jumping height was measured using a CMJ in centimeters with a flight time calculated using OptoJump (Microgate, Bolzano, Italy). The elevation of the center of the gravity was calculated for all jumps as the flight time (Ft) in seconds, applying the laws of ballistics, H = g(tv)2/8 (m), where H is the height and g is the gravitational acceleration [23]. The players were instructed to keep their hands on their hips and execute the landing without any leg flexion. Each test was executed 3 times, separated by 45 s of passive recovery in between. The best jump was recorded and used for analysis. The same criteria were used to assess CMJ right (CMJR) and CMJ left (CMJL).

#### 2.2.2. The Bilateral and Unilateral Horizontal Jump Tests

The jumping length was measured using an HJ test. To calculate the distance, a standard measuring tape (30 m M13; Stanley, New Britain, CT, USA, EEUU) was used. The players started with their toes behind the starting line on the ground and with their hands relaxed at their sides. After the signal of the investigator, each subject performed the jump, flexing and extending their legs as fast as they could, jumping as far as possible. After the landing, they had to maintain the position for 2–3 s. The distance was measured from the starting line to the heel [23]. Each HJ: HJ to the right (HJR), HJ to the left (HJL), and bilateral HJ (HJ) was performed 3 times with a 45 s rest in between. The best result was recorded and used for analysis.

#### 2.2.3. The 20 m Sprint

The running top speed of the tennis players was evaluated by a 20 m sprint time. The timing was performed using double-beam photoelectric cells (Witty, Micrograte, Bolzano, Italy). The timing gates were situated 1.5 m apart and at a height of 0.75 m. Each player was behind the starting line 0.5 m before the first marker and started the sprint at their own discretion (no external sign) [23]. Measurements were performed twice with a 2 min recovery between each attempt. A faster mark was recorded and used for analysis. The results were recorded in seconds (s) as the unit of measurement.

#### 2.2.4. The 180° COD Test

Players performed a 10 m sprint test with a 180° COD measured by photoelectric cells (Witty, Micrograte, Bolzano, Italy). The players sprinted from the starting line to the finish line, crossed the 5 m mark with the dominant and non-dominant leg, and turned 180° to sprint back to the start/finish line [23]. The 180° COD was repeated twice with the right leg (180CODR) and twice with the left leg (180CODL), and 2 min of between-repetition recovery was allowed. The best mark for each variant was recorded to calculate the mean time and statistical analysis.

### 2.3. Statistical Analysis

For statistical analysis, SPSS software (Version 28.0; SPSS Inc, Chicago, IL, USA) was used. First, the Shapiro–Wilk test was conducted to assess the normality of all variables. Once it was verified that the variables followed a normal distribution, a one-way repeated measures ANOVA was performed to identify the systematic bias between the means of asymmetry in performance variables. Lower-limb asymmetries were expressed as percentages (%) using the equation of Bishop et al. [4]. Pearson’s correlation coefficient was used to determine the correlation between limb asymmetries (%) and physical performance. In addition, the kappa coefficient was used to assess the consistency in the direction of asymmetry between tests, which can be interpreted as follows: poor (≤0), slight (0.01–0.20), fair (0.21–0.40), moderate (0.41–0.60), substantial (0.61–0.80), near perfect (0.81–0.99), and perfect [24]. A *p*-value of less than 0.05 was considered statistically significant.

## 3. Results

Table 1 provides data on the physical performance and asymmetries observed in female tennis players.

Table 2 shows Pearson’s correlations between the performance variables and the lower limb asymmetries. Significant relationships were found between CMJ asymmetry and the variables HJR (r = −0.47) and HJL (r = −0.44). In addition, significant relationships were found between HJ asymmetry and the variables CMJR (r = −0.60) and CMJL (r = −0.54), HJR (r = −0.64), HJL (r = −0.67), CMJ (r = −0.55), and HJ (r = −0.52). On the other hand, no significant relationships were found between COD asymmetry and the variables of jumping speed, speed, and change in direction.

**Figure 1:**
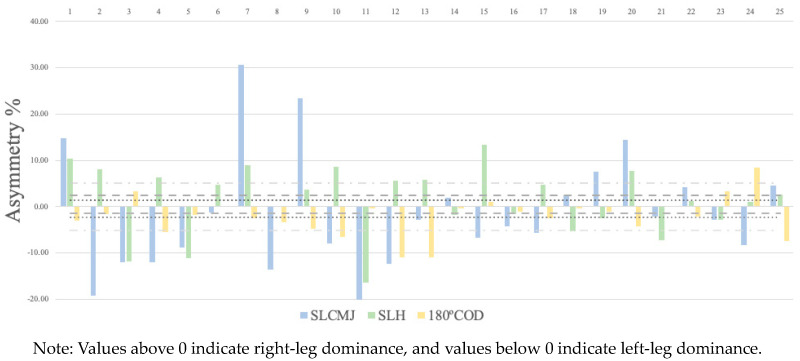
Individual asymmetry data for the single-leg countermovement jump (SLCMJ), single-leg horizontal jump (SLHJ), and 180° change of direction (180COD).

Table 3 presents the levels of agreement for the asymmetry scores obtained using the kappa coefficient. The results showed fair levels of agreement between the CMJ test and the 180COD test (−0.28) and slight levels between CMJ and HJ (0.09), and HJ and 180COD (−0.05). Figure 1 shows the discrepancies between the limbs for CMJ, HJ, and COD, highlighting the variability in both magnitude and direction of asymmetry in the study population.

Figure 1 shows how greater asymmetries are observed in the jumping variables than in the COD variables. We observed asymmetries greater than 10% in 10 subjects in CMJ, in 5 subjects in SLHJ, and in 2 subjects in the COD. The direction of the asymmetries in the variables favors the left leg (44 vs. 28).

## 4. Discussion

The primary objective of this study was to determine the correlation between interlimb asymmetries and physical performance variables in adolescent female tennis players.

Addressing lower-limb asymmetry in this population is highly important, as all movements are performed unilaterally, which can exacerbate the increase in asymmetries. These asymmetries can predispose athletes to injuries or a reduction in performance [25,26] Although this study was focused on adolescent female players, and it may limit the generalization of the findings, this sample was chosen because it represents an important stage in the development of strength and coordination. This study indicates that during adolescence, the neuromuscular systems become more refined, leading to more coordinated movement patterns, something essential to developing sport performance [27]. Also, during this stage, due to rapid physical changes, asymmetries tend to become more pronounced [28]. Furthermore, factors such as lateral dominance, motor preferences, and the specific demands of tennis can influence the asymmetries observed in these players [7].

The findings indicate significant negative correlations between HJ asymmetry and all bilateral and unilateral jump variables (r = −0.52 to −0.67). Moreover, negative correlations were found between CMJ asymmetry and unilateral HJs, both with the right leg (r = −0.47) and the left leg (r = −0.44). This means that as HJ asymmetry increases, performance in bilateral and unilateral jump tests (both bilateral and unilateral) decreases. Furthermore, the performance of unilateral HJs decreased with increasing CMJ asymmetry. The strong negative correlations suggest that significant imbalances between legs are associated with a lower jump performance. However, no significant relationships were found between COD asymmetry and any physical performance variables. Additionally, asymmetries between the jump and COD tests rarely favored the same side, indicating the task-specific nature of the asymmetry.

The SLCMJ showed the greatest asymmetries among the different tests (7.00 ± 10.50%), which is consistent with the findings of previous studies [7,9,11,18]. These data are fundamental, as they highlight possible imbalances in lower extremity function that could predispose tennis players to injury and reduce their performances. The CMJ showed significant negative correlations with unilateral HJs (HJR and HJL), which is interpreted as indicating that HJ performance decreases as CMJ asymmetry increases. This relationship provides information to understand how vertical power asymmetries affect lateral and forward movement skills, which are essential for tennis performance [29]. In contrast, no correlation was found between the vertical jumps studied (CMJR, CMJL, and CMJ), as observed in other studies and the 180COD [10,18].

Significant differences were observed in unilateral HJ asymmetries (6.03 ± 6.03%). This value is higher than those reported in similar studies of tennis players (4.14 ± 3.72%), [7,9,11] and (3.97 ± 4.18%) [18]. These asymmetries are particularly relevant in tennis because of the demands of rapid lateral movements, sudden directional changes, and rapid acceleration [30]. Horizontal jump asymmetry had a significant negative correlation with vertical and HJs in both unilateral and bilateral variants (r = −0.52 to −0.67). Greater asymmetry in the HJ test results in lower performance in all types of jumps evaluated in this study, contrary to Villanueva-Guerrero et al. [18]. Reducing asymmetries in the HJ should be a priority because doing so can improve vertical jumping performance, which is important for actions such as serves and smashes.

The 180COD test showed smaller asymmetries (1.93 ± 4.39) compared to both vertical and HJs, which is consistent with recent studies on tennis players [4,7,9,11,18,31] and female team-sports players [32]. This lower asymmetry in COD performance is logical given the nature of tennis, which requires frequent and rapid changes in direction between both legs, which should perform equally well to maintain agility and balance on the court [33]. In addition, no differences were found between the asymmetry in COD and performance variables, which means that COD asymmetry is not directly related to jump and sprint performance. One study only observed significant associations between COD asymmetry and HJ, but not between other variables, such as CMJ sprint performance [18]. This lack of correlation implies that COD performance is more dependent on overall coordination and the ability to decelerate and accelerate efficiently than on power and strength metrics that are more critical for jumps and sprints [34]. Consequently, while addressing asymmetries in jumping might improve explosive power, focusing on improving COD skills might improve COD speed and thus match performance, both of which are key to success in tennis.

Regarding the results of the Kappa’s analysis, only one agreement was found between the asymmetries in the CMJ and 180COD tests. There is a negative value between them, indicating an inverse relationship, which means that if CMJ asymmetry increases, the asymmetry in 180COD tends to decrease, or vice versa. These results may help professionals understand that training programs aiming to reduce asymmetry in one variable might not necessarily benefit from reduced asymmetry in another variable.

Significant differences in asymmetry values among different physical performance tests were identified among adolescent female tennis players. Several athletes exhibited more than 10% asymmetry in SLCMJ, highlighting notable lower-limb power imbalances. On the other hand, most players demonstrated asymmetries within the ±10% range in the SLH and 180COD tests, indicating better balance in these specific movements. These results are in accordance with women’ soccer [35] and male tennis players [18]. The asymmetries observed in the SLCMJ can be attributed to the unilateral nature of tennis, which involves the frequent and intense use of one leg over the other during serves, forehands, and backhands. Furthermore, players who use their forehand to cover most of the court may require higher acceleration to the forehand side [36] side. In addition, tennis involves fewer vertical movements, mainly in serves, than lateral (70%) and forward (20%) [29], leading to more pronounced asymmetries in the SLCMJ test. In contrast, the smaller asymmetries in the SLH and 180COD tests suggest that athletes have developed a relatively balanced ability to change direction and perform lateral movements, which are essential for effective court coverage in tennis. These lateral movements and CODs are more common in tennis, which could explain the improved symmetry performance in these tests. It is vital to communicate these results and findings to strength and conditioning coaches so they can implement training programs aimed at reducing limb asymmetries to below 10%, which is considered a low-risk range for injuries [11,25,26]. Furthermore, understanding gender differences, the impact of specific tennis skills and the use of advanced technology to assess asymmetries can provide more valuable insights for optimizing performance and preventing injuries.

Some limitations were found in this study. First, a larger sample size will provide more information and enhance the generalizability of the results. Second, since the study is cross-sectional, it only shows a specific moment in the season. It is recommended to conduct longitudinal studies to understand how asymmetries change during training and competition during a competitive season. Furthermore, the measurement tools used may have inherent limitations that could affect the accuracy of the collected data. Environmental or situational variables, such as weather conditions and the participants’ state at the time of evaluation, may also have influenced the results. Finally, it is important to note that these limitations may affect the interpretation of the findings; therefore, future studies are suggested to address these aspects and seek strategies to mitigate their impact.

## 5. Conclusions

The main findings of this study were that asymmetries in CMJ reduced performance in HJR and HJL, and asymmetries in HJ were associated with decreased performance in both bilateral and unilateral jumps (CMJR, CMJL, HJR, HJL, CMJ, and HJ). It did not find a significant correlation between asymmetry in 180COD and other performance variables, so it suggests that COD asymmetries do not influence jump performance or speed. It is necessary to periodically assess asymmetries in jumping variables to identify possible imbalances and thus implement specific training programs to help reduce these asymmetries. Since asymmetries are related to performance in jumping ability, their correction could have a positive impact on the physical performance of young female tennis players. These results underline the importance of further research and the evaluation of how asymmetries affect the performance of adolescent female tennis players. In this sense, it is suggested that coaches and physical trainers design specific programs that address these asymmetries, as their correction may be key to improving overall physical performance and reducing the risk of injury in these athletes.

## Figures and Tables

**Table 1:** Descriptive data for all physical performance tests.

Variable	Mean ± SD	Asymmetry (%)
CMJR (cm)	12.80 ± 3.30	7.00 ± 10.50
CMJL (cm)	12.96 ± 3.34	
HJR (cm)	138.12 ± 28.39	6.03± 6.03
HJL (cm)	136.72 ± 29.98	
180CODR (s)	2.91 ± 0.22	1.93 ±4.39
180CODL (s)	2.97 ± 0.21	
CMJ (cm)	25.30 ± 4.30	
HJ (cm)	173.20 ± 25.22	
20 m (s)	3.79 ± 0.17	

CMJR: unilateral countermovement jump with right leg. CMJL: unilateral countermovement jump with left leg. HJR: unilateral horizontal jump with right leg. HJL unilateral horizontal jump with left leg. 180CODR: 10 m shuttle sprint with one change in direction to the right. 180CODL: 10 m shuttle sprint with one change in direction to the left. 20 m: linear sprint of 20 m.

**Table 2:** Correlations between jumps, changes in direction tests, and asymmetry percentages.

Test	CMJ Asymmetry	HJ Asymmetry
CMJ (cm)	−0.23	−0.55 **
CMJR (cm)	−0.27	−0.60 **
CMJL (cm)	−0.25	−0.54 **
HJ (cm)	−0.19	−0.52 *
HJR (cm)	−0.47 *	−0.64 **
HJL (cm)	−0.44 *	−0.67 **
20 m (s)	−0.37	−0.09
180CODR (s)	0.26	0.07
180CODL (s)	0.30	0.16

CMJR: unilateral countermovement jump with right leg. CMJL: unilateral countermovement jump with left leg. HJR: unilateral horizontal jump with right leg. HJL unilateral horizontal jump with left leg. 180CODR: 10 m shuttle sprint with one change in direction to the right. 180CODL: 10 m shuttle sprint with one change in direction to the left. 20 m: linear sprint of 20 m; * Correlation is significant at the 0.05 level (bilateral).** Correlation is significant at the 0.01 level (bilateral).

**Table 3:** Kappa coefficients and descriptive levels of agreement of the asymmetries between the jumping speed and COD tests.

Test Comparison	Kappa Coefficient	Descriptor
CMJ-HJ	0.09	Slight
CMJ-180COD	−0.28	Fair
HJ-180COD	−0.05	Slight

## Data Availability

The data from this research can be made available by the corresponding author following a justified request. Due to privacy concerns, the data are not accessible to the public.
